# Supplementary material for: Adsorption Tuning of Polarity and Magnetism in AgCr2S4 Monolayer
Source: Materials (Basel). 2023 Apr 12;16(8):3058. doi: 10.3390/ma16083058 (PMC10140950; doi:10.3390/ma16083058)
Supplement: Supplementary file 1 [file materials-16-03058-s001.zip › materials-2286027-supplementary.pdf]

Supplementary Materials for

Adsorption tuning of polarity and magnetism in $AgCr_2S_4$ monolayer

Ranran Li,[1] Yu Wang,[1] Ning Ding,[1] Shuai Dong[1]* and Ming An[1]†

1 School of Physics, Southeast University, Nanjing 21189, China
* Correspondence: sdong@seu.edu.cn
† Correspondence: amorn@seu.edu.cn

Structure of the α and β phases of $AgCr_2S_4$

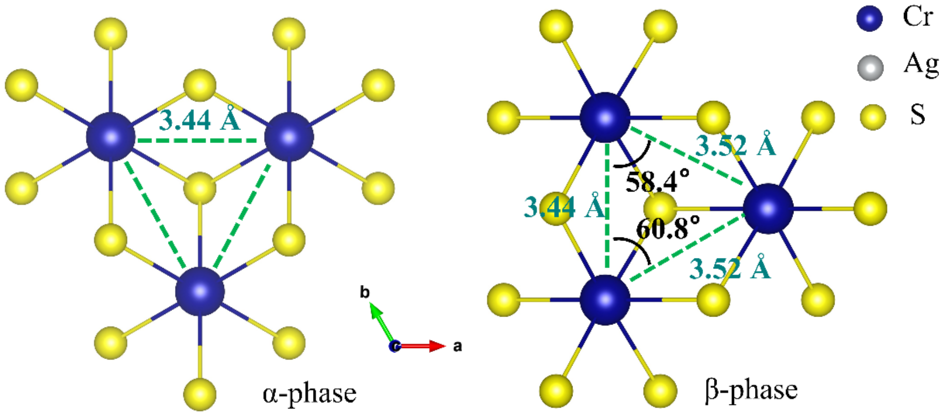

Figure S1. The detailed parameters for the in-plane structure of the asymmetric α phase (*P3m1*) and the centro-symmetric β phase (*C2/m*), respectively.

In addition to the subtle differences in the in-plane triangular lattice, the main difference between these two phases stems from the relative displacement of the intermediate Ag ion, as illustrated in Figure 2(a, b). Specifically, in the α phase, the AgS_2 tetrahedron inherited from parent bulk structure is well preserved. For β phase, the relative displacement of Ag results in a straight S-Ag-S bond between Ag and its upper and lower neighbor S ions. And the centralization of Ag leads to the centro-symmetry of the β phase. The lattice constants of these two structural phases are listed in Table S1.

Table S1. The lattice constants of the α and β phases, in units of Å.

	a	*b*
α phase	3.45	3.45
β phase	5.94	3.44

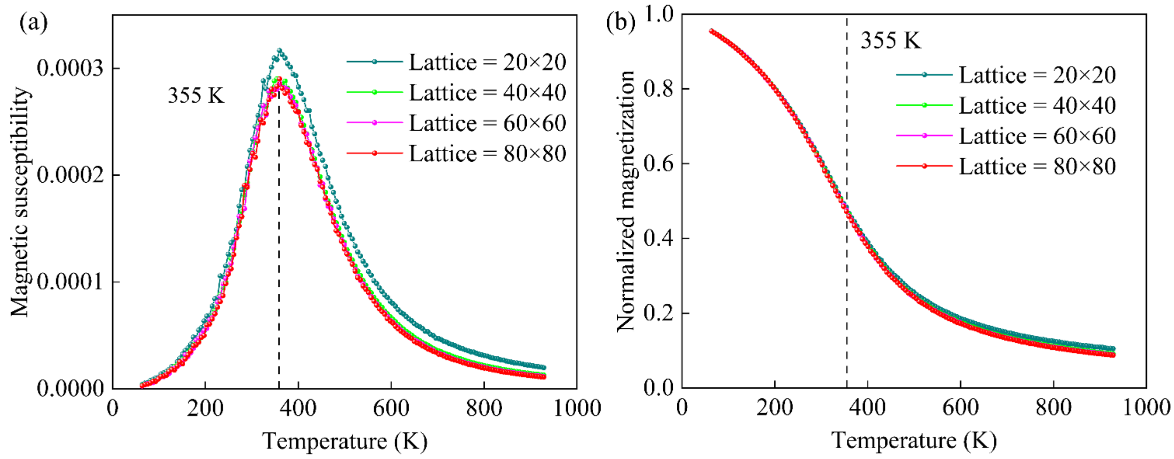

Figure S2. MC simulations of the magnetic susceptibility and normalized magnetization of $AgCr_2S_4$ monolayer at different lattice sizes as a function of temperature.

Adsorption energies

The adsorption energies (E_{ads}) were calculated as:

$$E_{ads} = E_{slab}^{ads} - E^{ads} - E_{slab}$$

E_{slab}^{ads}, E^{ads} and E_{slab} respectively represent the total energy of $AgCr_2S_4H$ system after hydrogen adsorption, the energy of H atom, and the energy of $AgCr_2S_4$ before adsorption. E_{ads} represents the adsorption energy. The value of adsorption energy is less than zero, indicating that the adsorption process is exothermic. The more heat released during adsorption, the more stable the adsorbed system ($AgCr_2S_4H$) is. It can be seen from Figure S3 that C2 site is the most ideal adsorption site with the lowest adsorption energy.

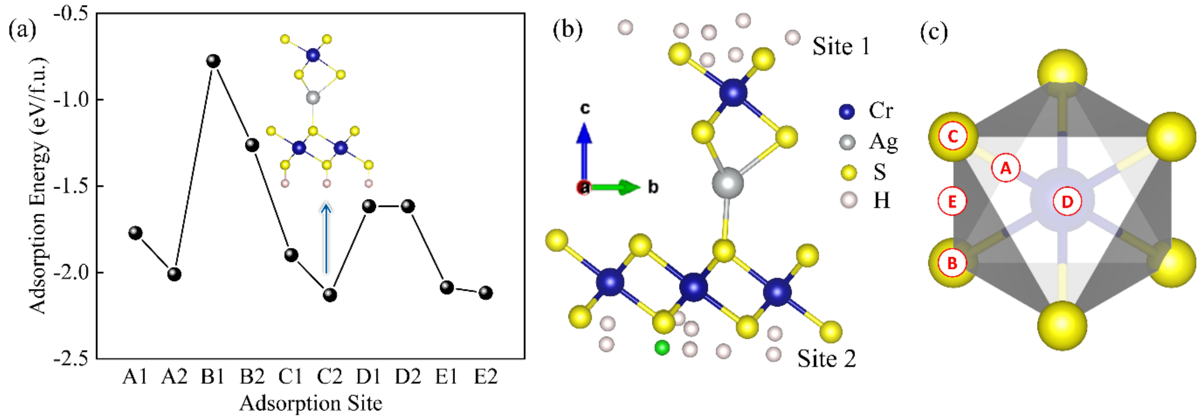

Figure S3. (a) Comparison of relative energy at different adsorption sites. (b) Side view of ten adsorption

H sites. Site 1 represents those close to Ag^+ ion, and Site 2 represents those relatively away from Ag^+. The lowest energy appears at C2 site, as denoted in green. (c) Top view of possible adsorption sites for hydrogen.

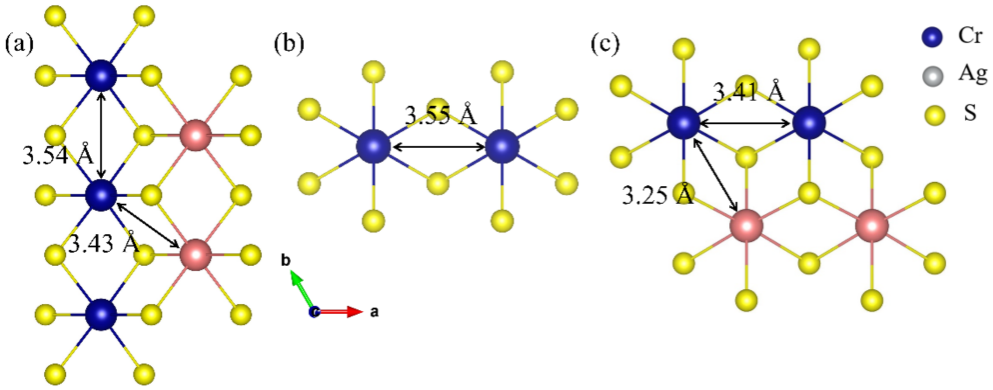

Figure S4. The in-plane distance between neighbor Cr ions. (a) Top view of CrS_2 layer in bulk $AgCrS_2$. (b) Ferromagnetic CrS_2 layer of freestanding $AgCr_2S_4H$. (c) Distorted triangular lattice of $AgCr_2S_4H$ under -3% in-plane biaxial strain. Cr ions with different spin orientations are distinguished by pink and blue spheres, respectively.

Mechanical stability and elastic properties of $AgCr_2S_4$

In this section, mechanical stability and elastic properties of $AgCr_2S_4$ are calculated using the strain-energy method [1]. First, to ensure the reliability of the calculation results, we calculated the Young's modulus of graphene. Our calculation result is 335 N/m, which agrees well with the experimental value (342 N/m) [2]. Then, further study was conducted on $AgCr_2S_4$ monolayer. A strain ranging from -1.5% to 1.5% was applied to the $AgCr_2S_4$ monolayer with an increment of 0.5%. The independent elastic constants of $AgCr_2S_4$ are listed in Table S2 [2].

Table S2. The in-plane elastic constants of single layer $AgCr_2S_4$ (in N/m).

	C_{11}	C_{12}	C_{22}	C_{66}
β-phase	117.729	22.928	124.403	39.587

The above results comply with the Born-Huang criteria of $C_{11}>0$, $C_{66}>0$, $C_{11}C_{22} > C_{12}^2$ [2,3], confirming that the material is stable.

To further study the mechanical properties of $AgCr_2S_4$, Young's modulus, shear modulus and Poisson's ratio are calculated and listed in Table S3 [4]. Compared with graphene (E = 342 N/m) and BN (E = 277 N/m) [5], $AgCr_2S_4$ has a smaller Young's modulus E, showing superior flexibility. The shear modulus of monolayer $AgCr_2S_4$ is about 44 N/m, while the shear modulus of boron nitride is 114 N/m [5]. $AgCr_2S_4$'s Poisson's ratio is 0.24, which is close to that of steel

(0.25). According to the these estimated elastic constants, it is reasonable to believe that $AgCr_2S_4$ is a soft and flexible material, which is similar to its three-dimensional counterpart [6].

Table S3. The Young's modulus *E* (N/m), shear modulus *G* (N/m), anisotropic shear factor (A^{SU}), and Poisson's ratio *v* of monolayer $AgCr_2S_4$.

E		*G*		A^{SU}	*v*
Max	Min	Max	Min	0.017	0.24
102.022	119.902	39.587	48.960		

The elastic anisotropy index A^{SU} (defined as the ratio of the normalized stiffness tensor C^V/C^R) is 0.017 for $AgCr_2S_4$ [4]. The schematic diagram of Young's modulus, shear modulus and Poisson's ratio are shown in Figure S5.

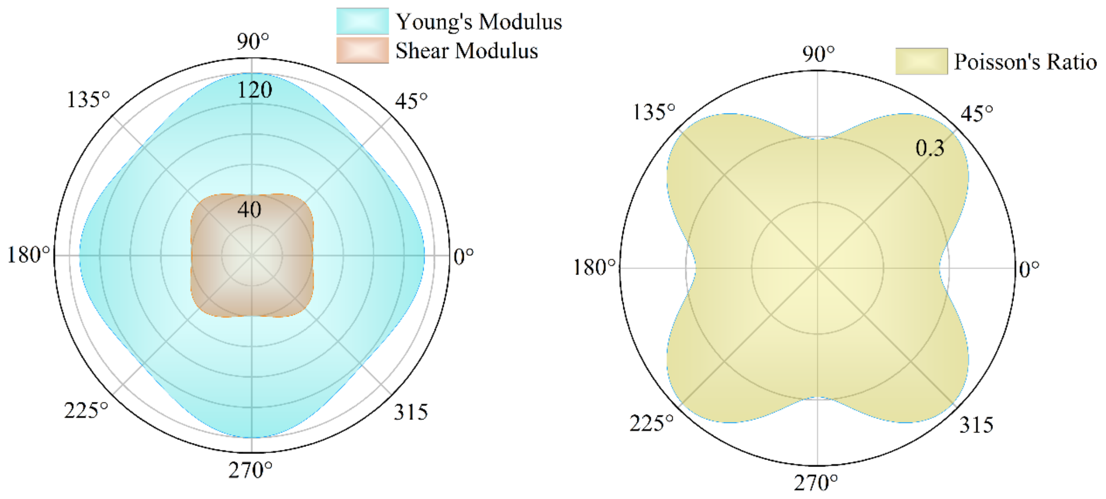

Figure S5. Orientation dependent in-plane Young's modulus *E* (θ), shear modulus *G* (θ), and Poisson's ratio *v* (θ) for $AgCr_2S_4$ respectively.

Table S4. Energy differences of the four in-plane collinear spin configurations of $AgCr_2S_4H_2$. Interlayer coupling is also considered, in which AFM interlayer coupling is expressed by subscript 1 and FM interlayer coupling is expressed by subscript 2. The total energy of the ground state (Z1-AFM) is used as the reference value, and the unit is meV/Cr.

FM	A-AFM	DS1-AFM	DS2-FM	Z1-AFM	Z2-FM	S1-AFM	S2-FM
100.02	125.57	60.78	60.63	0	0.37	61.68	62.39

References

1. Wang, V.; Xu, N.; Liu, J.-C.; Tang, G.; Geng, W.-T., Vaspkit: A user-friendly interface facilitating high-throughput computing and analysis using vasp code. *Comput. Phys. Commun.* **2021**, *267*, 108033.
2. Maździarz, M., Comment on 'the computational 2D materials database: High-throughput modeling and discovery of atomically thin crystals'. *2D Mate.* **2019**, *6* (4), 048001.
3. Born, M.; Huang, K., *Dynamical theory of crystal lattices*. Oxford University Press: USA**, 1954**, 120-154.
4. Li, R.; Shao, Q.; Gao, E.; Liu, Z., Elastic anisotropy measure for two-dimensional crystals. *Extreme Mech. Lett.* **2020**, *34*, 100615.
5. Falin, A.; Cai, Q.; Santos, E. J.; Scullion, D.; Qian, D.; Zhang, R.; Yang, Z.; Huang, S.; Watanabe, K.; Taniguchi, T., Mechanical properties of atomically thin boron nitride and the role of interlayer interactions. *Nat. commun.* **2017**, *8* (1), 15815.
6. Erkişi, A., Ab initio study on electronic and elastic properties of $AgCr_2S_4$. *Acta Phys. Pol. A* **2021**, *140* (3), 243-251.
